# Long‐term predictors of seizure outcome after anterior temporal lobectomy in unilateral hippocampal sclerosis: A 281‐patient cohort with mean 10‐year follow‐up

**DOI:** 10.1002/epd2.70139

**Published:** 2025-11-24

**Authors:** Thiago Pereira Rodrigues, Leonardo Favi Bocca, Elza Marcia Targas Yacubian, Mirian Salvadori Bittar Guaranha, Neide Barreira Alonso, Henrique Carrete Junior, Maria Helena Silva Noffs, Luis Otavio Caboclo, Jeana Torres Corso Duarte, Ricardo Silva Centeno

**Affiliations:** ^1^ Department of Neurology and Neurosurgery Universidade Federal de São Paulo São Paulo SP Brazil; ^2^ Hospital Israelita Albert Einstein São Paulo SP Brazil

**Keywords:** anterior temporal lobectomy, drug resistant epilepsy, hippocampal sclerosis, neurosurgery, temporal lobe epilepsy

## Abstract

**Objective:**

To identify long‐term predictors of seizure outcome after anterior temporal lobectomy (ATL) in a large, homogeneous cohort of patients with drug‐resistant temporal lobe epilepsy (TLE) and MRI‐defined unilateral hippocampal sclerosis (HS), all operated on by a single neurosurgeon with extended follow‐up.

**Methods:**

We retrospectively analyzed 281 consecutive patients with unilateral HS who underwent standardized ATL performed by the same senior neurosurgeon. All patients had at least two years of follow‐up (mean 10.8 ± 5.79 years). Clinical history, neuropsychological evaluation, long‐term video‐EEG monitoring, and 1.5T MRI constituted the preoperative dataset. Twenty‐one variables were assessed as potential predictors of seizure outcome. Kaplan–Meier survival curves and univariate log‐rank tests identified candidate predictors; variables with *p* < .10 were entered into a multivariate Cox regression model. Cognitive and quality‐of‐life outcomes were evaluated using standardized neuropsychological batteries and the ESI‐55 questionnaire.

**Results:**

At 10 years postoperatively, 62.6% of patients remained seizure‐free (Engel I). Univariate analysis identified seven factors associated with seizure freedom, including history of focal‐to‐bilateral tonic–clonic seizures, history of status epilepticus, presence of psychogenic non‐epileptic seizures, IED predominance or exclusivity in the operated lobe, ictal onset exclusively in the operated lobe, and a preoperative neuropsychological deficit confined to the operated temporal lobe. Multivariate analysis revealed three independent predictors of seizure outcome: history of status epilepticus (HR = 2.11; *p* = .002), ictal onset confined to the operated temporal lobe (HR = .57; *p* = .018), and preoperative neuropsychological deficit restricted to the operated temporal lobe (HR = .59; *p* = .040). Cognitive outcomes were generally stable; left ATL was associated with greater verbal memory decline. Quality‐of‐life improved significantly at 2‐year follow‐up (*p* < .001), with better outcomes among seizure‐free patients.

**Significance:**

In this large single‐surgeon cohort with one of the longest follow‐up durations reported, most patients with unilateral HS achieved durable seizure freedom after ATL. Status epilepticus, consistent ictal localization to the operated temporal lobe, and concordant preoperative neuropsychological deficit emerged as robust long‐term predictors. These findings reinforce the value of detailed presurgical evaluation—particularly ictal EEG concordance and neuropsychological lateralization—in optimizing surgical counseling, risk stratification, and patient selection.

**Key points**

**What is already known on this topic**

- The surgical outcome after anterior temporal lobectomy in hippocampal sclerosis is generally good; however, some patients continue to experience seizures despite optimal resection and antiseizure medication.

**What this study adds**

- The present study demonstrated, in a series of patients with pure unilateral sclerosis, that most patients became seizure‐free after anterior temporal lobectomy even during long‐term follow‐up. The absence of a history of status epilepticus, the presence of a temporal ictal onset zone in the operated temporal lobe for all epileptic events on VEEG, and preoperative neuropsychological evaluation in the functional deficit zone exclusively in the operated temporal lobe were independent predictors of seizure frequency in this series.

**How this study might affect research, practice, or the policy**

- Knowledge of the predictive factors associated with postoperative surgical outcomes is an important step in the preoperative decision‐making process and in improving the understanding of the causes of surgical failure. These predictive factors were not uniform in previous studies.

**Test yourself**

1. In this study, which preoperative factor was an independent predictor of long‐term seizure freedom after anterior temporal lobectomy (ATL) in patients with unilateral hippocampal sclerosis?
  - Age at epilepsy onset
  - History of febrile seizures
  - Preoperative neuropsychological deficit confined to the operated temporal lobe
  - Ictal dystonic posturing on video‐EEG
  - Duration of epilepsy
2. In this cohort, which video‐EEG characteristic was associated with a significantly better seizure‐free survival on multivariate analysis?
  - Presence of bilateral interictal epileptiform discharges
  - Ictal onset involving frontal and temporal lobes
  - Temporal ictal onset zone in the operated lobe for *all* recorded seizures
  - More than 80% of IEDs occurring in the contralateral temporal lobe
  - Presence of psychogenic non‐epileptic seizures (PNES)
3. Which of the following postoperative outcomes best reflects the long‐term Engel distribution reported in the study?
  - More than 90% of patients achieved Engel Class I
  - Approximately two‐thirds of the patients remained Engel Class I at long‐term follow‐up
  - Only 10% of patients showed meaningful seizure reduction
  - Engel classes remained unchanged from the first year to long‐term follow‐up
  - Most patients deteriorated to Engel Class IV after 5 years

*Answers may be found in the* *supporting information*

## 1 INTRODUCTION

Temporal lobe epilepsy (TLE) is the most common form of drug‐resistant epilepsy.^1^ The combination of TLE with temporal lobe abnormalities on Magnetic Resonance Imaging (MRI) is associated with drug‐resistant epilepsy in more than 70% of cases.^2^ For patients with drug‐resistant TLE, anterior temporal lobectomy (ATL) provides superior seizure control compared with continued medical therapy.^3^

Several surgical options are available for drug‐resistant TLE, including standard ATL and selective amygdalohippocampectomy (selAH). Although several studies suggest that anterior temporal lobectomy (ATL) and selective amygdalohippocampectomy (SAH) yield comparable long‐term seizure outcomes,^4^, ^5^ other reports indicate differences related to surgical technique, extent of resection, or patient selection, and the issue remains a matter of debate.^6^ Despite these favorable results, a significant proportion continue to experience seizures, underscoring the need to identify predictors of surgical outcome. Because postoperative seizure status is strongly correlated with quality of life,^7^ understanding which preoperative factors predict long‐term seizure control is critical for surgical counseling and patient selection.

Many previous studies have attempted to address this matter.^4^, ^8^, ^9^, ^10^, ^11^, ^12^, ^13^, ^14^, ^15^, ^16^, ^17^, ^18^, ^19^, ^20^, ^21^, ^22^, ^23^, ^24^, ^25^, ^26^, ^27^, ^28^ Several of them included diverse etiologies of TLE and considered the etiology of TLE as a possible predictive factor of postoperative seizure outcome.^8^, ^19^, ^20^, ^22^, ^23^, ^27^ Other authors have analyzed only patients with hippocampal sclerosis confirmed by postoperative histopathological evaluation,^11^, ^12^, ^13^, ^14^, ^15^, ^21^ or with preoperative MRI alterations compatible with hippocampal sclerosis in the context of TLE.^4^, ^16^, ^17^, ^24^, ^28^

However, even within the group of studies that included patients with TLE and preoperative MRI alterations compatible with hippocampal sclerosis, the results were not uniform. For instance, Aull‐Watschinger et al.^17^ and Srikijvilaikul et al.^24^ found no predictive factor of postoperative seizure outcome in conventional preoperative variables, whereas Janszky et al.,^16^ Mathon et al.,^28^ and Hemb et al.^4^ enumerated several. This scenario demonstrates that other factors, perhaps unevaluated, might influence postoperative epileptic status.

Recent investigations have emphasized the need to account for broader epileptogenic networks extending beyond the mesial temporal region, combining structural and functional connectivity analyses to refine prognostic models.^29^, ^30^, ^31^ Nevertheless, the ability to reliably predict, in the preoperative period, which patients will achieve long‐term seizure freedom remains limited.

Here, we report one of the largest single‐surgeon, long‐term cohorts of patients with drug‐resistant TLE and preoperative MRI findings of unilateral hippocampal sclerosis who underwent standard ATL at a Brazilian national epilepsy referral center. Our aim was to identify preoperative predictors of long‐term seizure outcomes, thereby contributing practical information for surgical decision‐making and patient counseling.

## 2 METHODS

### 2.1 Patients

This study was conducted in accordance with the Declaration of Helsinki and was approved by the institutional review board. We retrospectively reviewed patients with drug‐resistant temporal lobe epilepsy (TLE), diagnosed based on clinical history, neuropsychological assessment, long‐term video EEG monitoring, and MRI. Inclusion criteria were:

1. Unilateral MRI findings compatible with hippocampal sclerosis (HS), without other structural lesions.
2. Failure of at least two antiseizure medications (ASMs) at adequate doses, according to International League Against Epilepsy (ILAE) criteria.^32^
3. Undergoing standardized anterior temporal lobectomy (ATL) performed by the same senior neurosurgeon (RSC) between 2003 and 2019.
4. Minimum postoperative follow‐up of 2 years.

Invasive monitoring (ECoG/SEEG) was not included in this cohort. During the study period, invasive investigations were reserved for patients with non‐lesional MRI or with presurgical findings indicating bilateral/extratemporal involvement or substantial discordance. The present series, therefore, includes patients with MRI‐defined unilateral hippocampal sclerosis whose presurgical clinical, video EEG, and neuropsychological data were sufficiently consistent to proceed to anterior temporal lobectomy without invasive monitoring.

### 2.2 Video EEG monitoring

Long‐term noninvasive video EEG monitoring was performed using 32‐channel digital equipment (Ceegraph software, Bio‐Logic Systems Corp., Mundelein, IL, USA; and QP‐110AK, Nihon Kohden, Tokyo, Japan). Electrodes were placed according to the international 10–20 system, with additional temporal or sphenoidal electrodes as needed.

To record ictal events, ASMs were tapered or withdrawn at the physician's discretion (no standardized protocol applied). Interictal epileptiform discharges (IEDs) were visually assessed on 5‐min EEG samples per hour across the 24‐h monitoring period.

### 2.3 Neuroimaging

MRI scans were acquired using a 1.5T system (Magnetom Sonata, Siemens AG, Erlangen, Germany) with an eight‐channel head coil. The protocol included: sagittal T1‐weighted spin echo; coronal T2 fast spin echo, T2 FLAIR, and T1 inversion recovery; and axial T2 FLAIR and T2 gradient echo.

HS diagnosis was based on hippocampal atrophy evident on coronal T1 inversion recovery together with hyperintensity on coronal T2 or FLAIR. An experienced neuroradiologist (H.C.J.) reviewed all scans.

### 2.4 Surgical procedure

**FIGURE 1:**
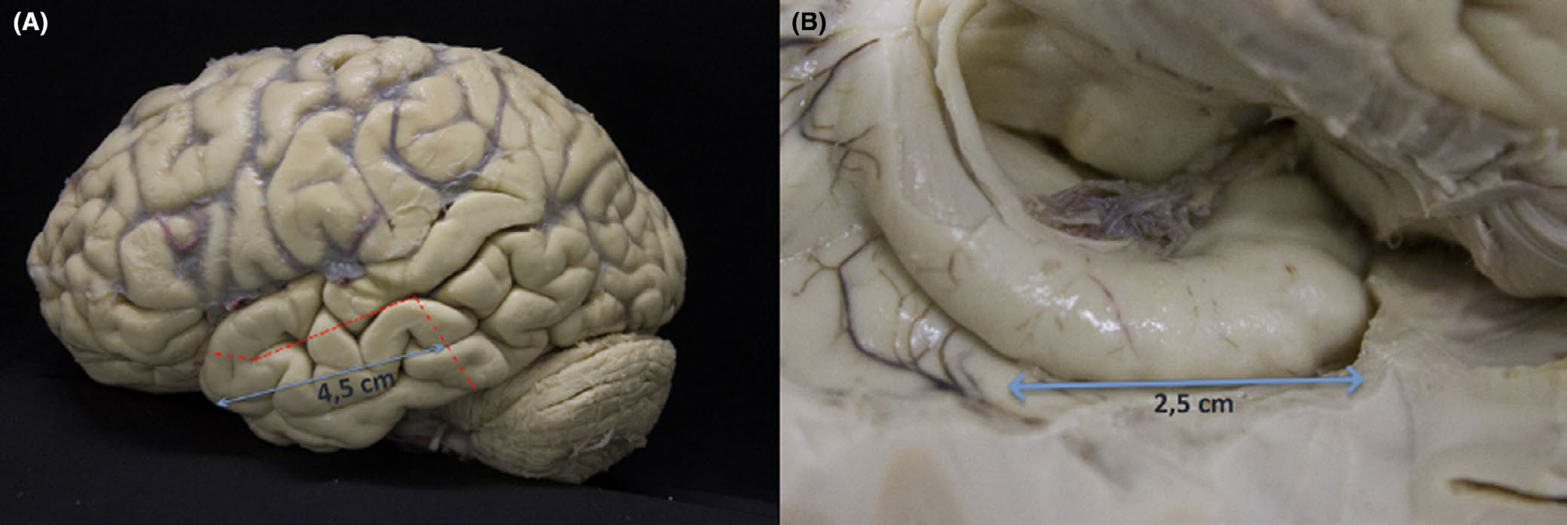
Anatomical temporal lobe dissection. (A) The red dotted line shows the limits of the neocortex, which was the anterior 4.5 cm portion of the temporal lobe. (B) Right mesial temporal lobe dissection exposing intraventricular view of the right hippocampus. We performed an en bloc resection of the anterior 2.5 cm hippocampal portion.

All patients underwent standard ATL by the same senior neurosurgeon (R.S.C) (Figure 1):

- En bloc resection of the temporal neocortex, including its anterior 4.5 cm.
- Piecemeal resection of the uncus, amygdala, and entorhinal cortex.
- En bloc resection of the anterior hippocampus (anterior 2.5 cm or more).

### 2.5 Neuropsychological testing

Patients underwent neuropsychological tests to assess cognitive functions, such as attention, executive functioning, memory, language, and intellectual level. All tests were administered preoperatively to evaluate surgical candidates and replicated in the postoperative period. The intelligence quotient (IQ) was estimated using the Wechsler Adult Intelligence Scale‐Revised (WAIS‐R).^33^ Logical memory and immediate and delayed recall were used to evaluate visual memory. In addition, the Rey‐Osterrieth Complex Figure Test,^34^ immediate and delayed recall, were used to assess spatial memory, and the Rey Auditory Verbal Learning Test (RAVLT) was used to assess verbal learning. For attention and executive functioning, the Trail Making Test (A and B), Digit Span (WAIS), and Stroop Color Word Interference Test were used. For language, the Controlled Oral Word Association Test (FAS version), Animal Fluency, Boston Naming Test (BNT), and Vocabulary (Wechsler Adult Intelligence Scale‐III) tests were used.

In the clinical routine, the epilepsy surgery multidisciplinary team analyzes the entire preoperative neuropsychological evaluation data and accordingly defines a functional deficit area. In this study, for statistical analyses, attention scores, executive functioning scores, and language scores were included to determine whether the functional deficit zone included the frontal lobes. Accordingly, the Rey Auditory Verbal Learning Test (RAVLT) was used to evaluate verbal learning and memory and, therefore, determine the involvement of the dominant temporal lobe. The Rey‐Osterrieth Complex Figure was used for visual memory and non‐dominant temporal lobe evaluation. In statistical analyses, temporal lobe dominance was classified as dominant and non‐dominant on the left and right sides, respectively.

The patients were segregated into two groups based on the following preoperative neuropsychological analysis: patients with a functional deficit area present exclusively in the operated temporal lobe, patients with other lobe functional deficits, and/or patients with no functional deficits (more extensive functional deficit zone or no functional deficit).

A postoperative neuropsychological evaluation was performed, similar to the preoperative evaluation. The timing of postoperative neuropsychological testing was not uniform, but we included only patients with postoperative neuropsychological evaluations conducted at least 1 year after surgery. Postoperative neuropsychological assessments were conducted at variable time points, depending on clinical follow‐up and staff availability. Because not all patients were evaluated exactly 2 years after surgery, the last available postoperative assessment for each patient was used for analysis.

### 2.6 Quality‐of‐life assessment

Health‐related quality of life (HRQOL) was assessed using the epilepsy surgery inventory 55 (ESI‐55) preoperatively and at the 2‐year postoperative follow‐up. Authors assessed 10 domains of the ESI‐55: health perceptions, energy/fatigue, overall quality of life, social function, emotional well‐being, cognitive function, physical function, and role limitations due to emotional problems, memory problems, and physical problems. These domains were weighted and summed to generate the overall HRQOL score.^35^

### 2.7 Analyzed variables

Analysis assessed 21 variables for possible prognostic implications: Laterality of surgery; Age at operation; Age at epilepsy onset; Duration of epilepsy prior to surgery; History of febrile seizures; History of perinatal complications; History of prior central nervous system infection; History of prior brain trauma (with loss of consciousness); First‐degree relative with epilepsy; History of ascending epigastric aura; Preoperative seizure frequency (grouping patients into those who had more than one seizure per week [defined as high frequency] and those with less than or equal to one seizure per week [defined as low frequency]); History of two or more focal seizures evolving to bilateral tonic–clonic seizures (only considered patients with focal seizures evolving to bilateral tonic–clonic during ASM use); Histopathological type of hippocampal sclerosis; History of status epilepticus; Ictal limb dystonic posture on video EEG; Presence of psychogenic non‐epileptic seizures (PNES) on video EEG; More than 80% interictal epileptic discharges (IEDs) on video EEG in the operated temporal lobe; 100% IEDs on video EEG in the operated temporal lobe; Presence of temporal ictal onset zone in the operated temporal lobe in all video EEG epileptic events; Preoperative neuropsychological evaluation (whether the functional deficit area was exclusively in operated temporal lobe or not); and Preoperative IQ.

Predictor variables were chosen a priori based on previously published studies on ATL outcomes and on our center's clinical experience. Semiologic features such as epigastric (viscerosensory) aura and ictal dystonic posturing were included because of their recognized lateralizing value.^16^, ^36^, ^37^ Duration of epilepsy was analyzed given that, in our cohort, presurgical latency was considerably longer than that described in most series from developed countries—an aspect that may influence long‐term outcomes and reflects real‐world practice in resource‐limited settings.

### 2.8 Statistical analysis

Statistical analysis was performed using the SPSS software (SPSS Inc., 20.0, Chicago, IL, USA, 2008). To assess the treatment outcome on survival curves, seizure‐free status was defined as the absence of any epileptic events, including auras. Additional survival curves were plotted for patients who were free of impaired awareness seizures (Engel 1A + 1B). Additionally, postoperative outcomes based on the time from surgery (2, 5, and 10 postoperative years) were analyzed. In the final scenario, the Engel seizure classification was used.

Categorical variables were described as numbers of cases and percentages, and for group comparisons, either the chi‐squared test or Fisher's exact test was used, as appropriate. Quantitative variables are presented as mean ± standard deviation, and group comparisons were performed using unpaired *t*‐tests. Survival curves were estimated using the Kaplan–Meier method and compared using log‐rank tests. Seizure outcome was analyzed longitudinally using Kaplan–Meier survival curves, with the event defined as postoperative seizure recurrence (including auras). Patients were censored at their last available follow‐up, allowing estimation of seizure‐free survival across 2, 5, and 10 years after surgery and beyond.

To identify the risk factors for seizures, univariate (log‐rank) and multivariate Cox regression analyses based on survival curves were performed. All variables with a *p*‐value <.10 in univariate analysis were entered into the multivariate Cox regression model. This threshold was deliberately chosen, rather than the stricter cutoff of *p* < .05, to reduce the risk of overlooking potentially relevant predictors. By adopting this inclusive criterion, we aimed to balance the risk of type II error with the need to maintain a parsimonious model and avoid overfitting.

Statistical significance was set at *p* < .05.

For cognitive outcomes, we compared pre‐ and postoperative neuropsychological scores using the Reliable Change Index (90% confidence interval).^38^

## 3 RESULTS

This study included 281 patients (122 men and 159 women). The clinical features of the patients are shown in Table 1. The postoperative outcomes using the Engel seizure classification are shown in Table 2.

**TABLE 1:** Clinical features of 281 patients.

Parameter	Value (%)
Left temporal lobe epilepsy	153 (54.4)
History of ascending epigastric aura	137 (48.8)
First‐degree relative with epilepsy	40 (14.2)
History of febrile seizures	79 (28.1)
History of perinatal complications	8 (2.8)
History of prior brain trauma (with loss of consciousness)	27 (9.6)
History of prior central nervous system infection	18 (6.4)
History of focal evolving to bilateral tonic–clonic seizures (≥2 events)	191 (68)
History of status epilepticus	68 (24.2)
Preoperative Focal Impaired awareness seizures frequency (>4 per month/4 or less per month)	108 (38.4)
Preoperative neuropsychological evaluation with functional deficit zone exclusively in the operated temporal lobe	79^a^ (28.1)
Histopathological type of HS	
Type 1	68/81 (84%)
Type 2	7/81 (8.6%)
Type 3	2/81 (2.5%)
Type no‐HS	4/81 (4.9%)
Presence of psychogenic non‐epileptic seizures on VEEG	11^b^ (4.8)
Ictal limb dystonic posture in VEEG	122^b^ (43.4)
Presence of temporal ictal onset zone in the operated temporal lobe for all epileptic events on VEEG	215^b^ (80.2)
More than 80% of IEDs in operated temporal lobe on VEEG	229^b^ (82.1)
100% IEDs in operated temporal lobe on VEEG	146^b^ (54.5)

Parameter	Mean (SD)
Age at operation	36.74 (11.51)
Age at epilepsy onset	14.25 (8.78)
Duration of epilepsy	22.43 (11.91)
Intelligence quotient	81.01 (10.44)

Abbreviations: IED, interictal epileptiform discharge; VEEG, video‐electroencephalography.

^a^ We analyzed the preoperative neuropsychological evaluations of the 249 patients.

^b^ We analyzed VEEG data from 268 patients.

**TABLE 2:** Postoperative Engel class.

	1 year		5 years		10 years		15 years	
	*n*	%	*n*	%	*n*	%	*n*	%
Engel 1A	183	65.1	131	46.6	92	51.4	34	49.3
Engel 1B	20	7.1	23	8.2	18	10.1	8	11.6
Engel 1C	0	0	19	6.8	21	11.7	11	15.9
Engel 1D	3	1.1	6	2.1	3	1.7	3	1.1
Engel II	56	19.9	37	13.2	22	12.3	9	13
Engel III	17	6.0	13	4.6	7	3.9	4	5.8
Engel IV	2	.7	2	.7	1	.6	0	0
Lost follow‐up	0	0	15	5.3	15	8.4	0	0
Total	281		246		179		69	

The mean follow‐up duration was 10.8 years (±5.79). In the univariate analysis (Table 3), there were seven predictive factors for Engel class 1 in the Kaplan–Meier curves: history of focal seizures evolving to bilateral tonic–clonic seizures (*p* < .001), history of status epilepticus (*p* < .001), presence of psychogenic non‐epileptic seizures on video EEG (*p* = .004), more than 80% of IEDs on video EEG (VEEG) in the operated temporal lobe (*p* = .067), 100% IEDs on VEEG in the operated temporal lobe (*p* = .002), presence of temporal ictal onset zone in the operated temporal lobe for all epileptic events on VEEG (*p* < .001), and preoperative cognitive deficit in the operated temporal lobe (*p* = .001).

**TABLE 3:** Univariate analysis of variables.

Qualitative variables (log‐rank test)	Average time to event ± SD, year	*p*‐Value
Left temporal lobe epilepsy (yes/no)	11.2 ± .61/11.1 ± .67	.895
History of febrile seizures (yes/no)	11.11 ± .85/11.19 ± .53	.985
First‐degree relative with epilepsy (yes/no)	11.53 ± 1.19/11.12 ± .49	.746
History of ascending epigastric focal aware seizures (yes/no)	11.71 ± .63/10.67 ± .64	.287
Preoperative Focal Impaired awareness seizures frequency (>4 per month/4 or less per month)	10.67 ± .75/11.45 ± .56	.446
Ictal dystonia (yes/no)	10.73 ± .69/11.50 ± .59	.366
History of focal evolving to bilateral tonic–clonic seizures (yes/no)	10.00 ± .56/13.76 ± .66	<.001
Status epilepticus (yes/no)	6.50 ± .91/12.67 ± .48	<.001
Preoperative neuropsychological evaluation with functional deficit zone exclusively in the operated temporal lobe (yes/no)	13.54 ± .72/10.01 ± .59	.001
Histopathological ILAE HS type (type 1/type 2)	12.53 ± 1.19/12.12 ± 1.49	.705
Presence of psychogenic non‐epileptic seizures on VEEG (yes/no)	7.46 ± 2.1/11.56 ± .46	.041
More than 80% of IEDs on VEEG in operated temporal lobe (yes/no)	11.76 ± .49/9.51 ± 1.13	.067
100% IEDs on VEEG in operated temporal lobe (yes/no)	12.66 ± .57/9.78 ± .71	.002
History of perinatal complications (yes/no)	11.12 ± 2.68/11.17 ± .46	.998
History of prior brain trauma (yes/no)	8.49 ± 1.40/11.40 ± 10.48	.088
History of prior central nervous system infection (yes/no)	10.83 ± 1.82/11.19 ± .46	.860
Presence of temporal ictal onset zone in operated temporal lobe for all epileptic events on VEEG (yes/no)	12.19 ± .49/8.00 ± 1.08	<.001

Quantitative variables (*t*‐test)	Seizure‐free (*n* = 176)	Seizure (*n* = 105)	*p*‐Value
Age at surgery (years)	36.66 ± 11.41	36.89 ± 11.71	.873
Age at epilepsy onset (years)	14.40 ± 8.67	13.98 ± 9.01	.697
Duration of epilepsy (years)	22.26 ± 11.84	22.71 ± 12.08	.756
IQ^a^	81.31 ± 10.23	80.52 ± 10.81	.558

^a^ We analyzed 249 preoperative neuropsychological evaluations.

In the multivariate analysis (Table 4), only three factors remained statistically significant: history of status epilepticus (*p* = .002), presence of a temporal ictal onset zone in the operated temporal lobe for all epileptic events on VEEG (*p* = .018), and preoperative neuropsychological evaluation with a functional deficit zone exclusively in the operated temporal lobe (*p* = .04). None of the variables that entered the multivariate model with univariate *p*‐values between .05 and .10 remained significant. Consequently, using a stricter entry criterion (*p* < .05) resulted in the same independent predictors, confirming the robustness of the model.

**TABLE 4:** Multivariate analysis (Cox Regression).

Variables	Multivariate analysis, cox regression model	
	Hazard ratio (95% CI)	*p*‐Value
History of focal evolving to bilateral tonic–clonic seizures (yes/no)	1.385 (.769–2.496)	.278
Status epilepticus (yes/no)	2.113 (1.322–3.377)	.002
Preoperative neuropsychological evaluation with functional deficit zone exclusively in the operated temporal lobe (yes/no)	.594 (.374–.945)	.040
Presence of psychogenic non‐epileptic seizures on VEEG (yes/no)	1.661 (.784–3.519)	.185
More than 80% of IEDs on VEEG in operated temporal lobe (yes/no)	1.030 (.598–1.773)	.916
100% IEDs on VEEG in operated temporal lobe (yes/no)	.698 (.428–1.139)	.150
History of prior brain trauma (yes/no)	1.514 (.818–2.802)	.187
Presence of temporal ictal onset zone in operated temporal lobe for all epileptic events on VEEG (yes/no)	.574 (.363–.909)	.018

**FIGURE 2:**
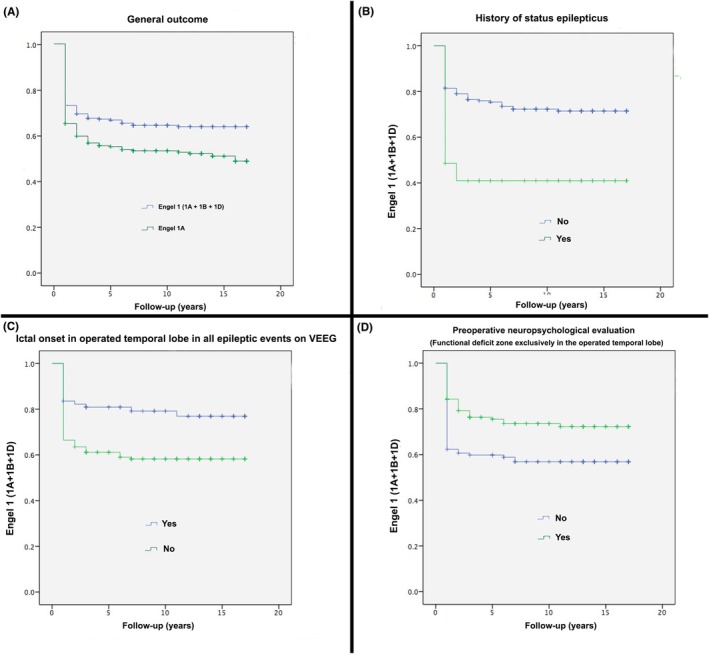
Kaplan–Meier survival curves. Survival curve of all patients (A), according to history of status epilepticus (B), ictal onset in operated temporal lobe in all epileptic events on VEEG (C), and presence of functional deficit zone exclusively in the operated temporal lobe (D).

The frequencies of seizure‐free (Engel 1A + Engel 1B) patients at the end of 1, 2, 5, and 10 postsurgical years were 71.9%, 68.2%, 64.4%, and 62.6%, respectively. Segregated data of the seizure‐free survival curve according to statistically significant multivariate analysis factors is shown in Figure 2.

### 3.1 Cognitive outcomes

When comparing preoperative and postoperative evaluations, most patients showed stability in cognitive performance across domains.

#### 3.1.1 Intelligence quotient (IQ)

Among patients undergoing right ATL (*n* = 91), IQ worsened in 3, remained stable in 71, and improved in 17 cases. Among left ATL patients (*n* = 111), IQ worsened in 7, was unchanged in 95, and improved in nine cases. These differences were not statistically significant (*p* = .349 for right ATL; *p* = .661 for left ATL). A trend toward improvement was observed in right ATL patients (*p* = .050).

#### 3.1.2 Verbal memory

After right ATL (*n* = 85), verbal memory worsened in three patients, was unchanged in 78, and improved in 4. After left ATL (*n* = 99), verbal memory worsened in 18, was unchanged in 79, and improved in two patients. Decline in verbal memory was significantly more frequent after left ATL (*p* = .005).

#### 3.1.3 Visuospatial memory

In the right ATL group (*n* = 87), visuospatial memory worsened in seven patients, remained stable in 72, and improved in 8. In the left ATL group (*n* = 107), visuospatial memory worsened in 16, was unchanged in 84, and improved in 7. No significant differences were observed (*p* = .187 for right ATL; *p* = .806 for left ATL).

### 3.2 Quality‐of‐life outcomes

Preoperative and 2‐year postoperative follow‐up HRQOL evaluations were performed for 107 patients. Among them, 72.9% improved (78 of 107), 15.9% remained unchanged, and 11.2% (12 of 107) reported worsening of HRQOL after surgery.

Of the 12 patients whose HRQOL worsened after surgery, six developed symptoms of depression in the postoperative period, two experienced a poor seizure outcome (Engel IV), one had degenerative lumbar spine disease that prevented him from walking (not related to the epilepsy surgery itself), one had a visual field deficit that reduced her HRQOL (quadrantanopsia), one had documented cognitive worsening, and one was diagnosed with an HIV infection and consequent opportunistic infections.

In the preoperative period, the mean overall HRQOL score was 61.44 (±19.66) and in the postoperative period it was 78.52 (±18.64). This difference was statistically significant (*p* < .001).

In terms of work status, 22.43% (*n* = 24) began to work after surgery. Only 3.7% (*n* = 4) of patients stopped working after surgery.

Our analysis demonstrated a significant association between postoperative seizure outcome and HRQOL improvement. Patients who remained seizure‐free (Engel I) reported higher rates of HRQOL improvement compared with those who continued to experience seizures (*p* = .0027 when auras were included; *p* = .0087 when auras were excluded).

## 4 DISCUSSION

In this large single‐center series of 281 patients with unilateral hippocampal sclerosis (HS) who underwent anterior temporal lobectomy (ATL), we report long‐term seizure, cognitive, and quality‐of‐life outcomes after a mean follow‐up of 10.8 years. Independent predictors of seizure freedom were: (1) absence of a history of status epilepticus; (2) consistent ictal onset restricted to the operated temporal lobe, and (3) presence of a preoperative neuropsychological deficit confined to the resected temporal lobe. Overall seizure‐free rates (63% at 10 years) are consistent with the long‐term outcomes reported in previous cohorts.^4^, ^7^, ^8^, ^9^, ^10^, ^11^, ^12^, ^13^, ^14^, ^15^, ^16^, ^17^, ^18^, ^19^, ^20^, ^21^, ^22^, ^23^, ^24^, ^25^, ^26^

A history of status epilepticus^27^ and a history of focal evolution to bilateral tonic–clonic seizures^4^, ^15^, ^39^ were associated with worse seizure outcomes in previous studies. In the Mathon et al. series,^27^ a history of status epilepticus was a predictive factor for postoperative seizure control, but the occurrence of preoperative generalized seizure (at least one generalized seizure) was not. Hemb et al.^4^ found that patients with preoperative generalized tonic–clonic seizures had worse postoperative seizure control; however, in that series, the occurrence of status epilepticus was not measured. Janszky et al.^15^ performed univariate analyses at 6 months, 2 years, 3 years, and 5 years after surgery. Only at the 2‐years postoperative univariate analyses, the presence of secondarily generalized tonic–clonic seizures was associated with worse postoperative seizure control.

In our patients, both factors were predictors in the univariate analysis; however, in the multivariate analysis, only a history of status epilepticus remained statistically significant. Furthermore, if we did not analyze the history of status epilepticus in our patients, the history of focal seizures evolving to bilateral tonic–clonic seizures would be a predictor, even in the multivariate model. Both frequent focal seizures evolving to bilateral tonic–clonic seizures and status epilepticus suggest the presence of a broader epileptogenic network rather than a purely focal lesion, potentially explaining their impact on surgical outcomes. In addition, status epilepticus itself can induce neuronal lesions that are distant from the temporal primary focus.

The relationship between ictal scalp EEG and outcomes in terms of seizure control after anterior temporal lobectomy has been analyzed in the literature. Assaf and Ebersole^40^ showed that preoperative ictal EEG patterns of hippocampal origin (type 1) were associated with freedom from seizures in all patients in their series. Sirin et al.^41^ also demonstrated that ictal onset pattern correlates with seizure outcomes after surgery. In our study, the patients were divided based on whether all epileptic events registered in the long‐term noninvasive video EEG monitoring were in the operated temporal lobe. The group of patients with all epileptic events on video EEG in the operated temporal lobe had a better outcome in terms of postoperative seizure control.

The value of preoperative neuropsychological assessment as a predictive factor for postoperative seizure status has been tested in previous studies. Chelune et al.^42^ found that patients who continued to have seizures following temporal lobe surgery for epilepsy had a small but statistically significant lower preoperative IQ score. Potter et al.^43^ evaluated the relationship between preoperative neuropsychological assessments and seizure control. They found that patients with higher verbal/language scores and lower non‐verbal memory scores had better postoperative seizure control. In this series, testing scores for attention and executive functioning, language, verbal memory, verbal learning, visual memory, and spatial memory were compared at pre‐ and postoperative moments. The results showed that in patients whose functional deficit zone was completely removed, the prognosis in terms of postoperative seizure control was better than that in patients who had either a more extensive functional deficit zone or a normal preoperative neuropsychological evaluation. In this series of patients, preoperative IQ scores were not a statistically significant predictor (in previous studies that found this association, the number of patients was much higher).

By analyzing preoperative and postoperative neuropsychological evaluations, patients who underwent right anterior temporal lobectomy showed a tendency for improvement in IQ scores when compared with patients who underwent surgery on the left side (*p* = .05). In addition, compared with patients operated on the right side, those operated on the left side had a decline in verbal memory (*p* = .005). Resection of the dominant temporal lobe correlated with greater verbal memory decline, whereas resections of the non‐dominant temporal lobe correlated with greater visual memory impairment, although with less consistency.^44^ In this series, patients who underwent right ATL had no worsening of visual memory, and patients who underwent surgery on the left side had a significant decline in verbal memory (*p* = .005).

Most patients in this study showed a significant improvement in HRQOL. Among the patients with worsening HRQOL, the main cause was depression (6 of 12). Previous studies have shown no association between preoperative depression and postoperative seizure control.^28^, ^45^ Furthermore, this series of patients was not systematically assessed, either preoperatively or postoperatively, for psychiatric disorders, that is, evaluations were performed on a clinical needed basis. However, we found that symptoms of depression were highly prevalent in patients with a significant decline in their postoperative HRQOL.

The observed correlation between seizure freedom and HRQOL improvement reinforces the well‐established link between successful seizure control and enhanced psychosocial functioning. In our cohort, patients who achieved complete seizure freedom, even when auras were included, exhibited markedly greater improvement in HRQOL scores. These findings are consistent with prior reports highlighting seizure outcome as a determinant factor of postoperative quality‐of‐life gains.

The present study represents, to our knowledge, one of the largest single‐surgeon, long‐term cohorts of anterior temporal lobectomy in patients with MRI‐defined unilateral hippocampal sclerosis. Its principal strengths include the homogeneity of the sample, the extended duration of follow‐up, and the integrated assessment of seizure, cognitive, and quality‐of‐life outcomes.

Several limitations should also be acknowledged. The retrospective design inherently limits data completeness, particularly regarding histopathological information. The timing of postoperative neuropsychological evaluations was not uniform, as there were periods during which specialized personnel were unavailable; although we used each patient's last available assessment to reflect long‐term cognitive status, this variability may have introduced some heterogeneity. The inclusion of a broad range of potential predictors may have increased model complexity, and certain variables—such as PNES—are more closely related to psychosocial factors than to seizure generation. Nonetheless, their inclusion allowed for a more comprehensive and comparative analysis consistent with prior literature. All MRI studies were performed on a 1.5T scanner, which aligned with the clinical standard during most of the study period but may have reduced sensitivity to subtle or dual pathologies. Finally, patients who required invasive intracranial monitoring were excluded; while this ensured a homogeneous MRI‐positive cohort, it limits generalizability to non‐lesional or presurgically discordant temporal lobe epilepsy.

Although most predictors identified have been previously described, the present study strengthens the existing evidence by validating these findings in a large, homogeneous, and consistently operated cohort with one of the longest follow‐up periods reported. The uniform surgical technique, long‐term outcome data, and combined analysis of seizure, cognitive, and quality‐of‐life measures offer a comprehensive and real‐world view of anterior temporal lobectomy results in a middle‐income healthcare context.

### 4.1 Clinical implications

These findings reinforce that status epilepticus, consistent ictal onset localization, and concordant neuropsychological deficit are clinically relevant predictors that can guide surgical counseling. Our results add to the growing body of evidence suggesting that patients with broader epileptogenic networks or atypical neuropsychological profiles may require closer follow‐up and may benefit less from ATL alone.

## 5 CONCLUSION

In this large single‐center cohort with long‐term follow‐up, most patients with unilateral hippocampal sclerosis achieved durable seizure freedom after anterior temporal lobectomy. Independent predictors of favorable outcomes included absence of status epilepticus, consistent ictal onset localized to the operated lobe, and a preoperative neuropsychological deficit confined to the resected temporal lobe. These findings highlight the importance of integrating detailed seizure history, prolonged video EEG, and neuropsychological evaluation into preoperative assessment. This study provides one of the largest single‐surgeon experiences reported to date and reinforces the value of comprehensive preoperative evaluation in optimizing surgical counseling and patient selection.

## AUTHOR CONTRIBUTIONS

T.P.R. designed the study, analyzed the data, and drafted the manuscript. L.F.B., M.S.B.G., N.B.A., H.C.J., and M.H.S.N. contributed to data collection and clinical evaluations. L.O.C. and J.T.C.D. contributed to data analysis and critical manuscript revision. E.M.T.Y. and R.S.C. supervised the project and critically revised the final version. All authors approved the final manuscript.

## FUNDING INFORMATION

This research received no external funding. The study was conducted using institutional resources from the Universidade Federal de São Paulo.

## CONFLICT OF INTEREST STATEMENT

The authors declare that they have no commercial or financial conflicts of interest that could be construed as a potential conflict of interest.

## Supporting information


Appendix S1.

## Data Availability

De‐identified data supporting the findings of this study are available from the corresponding author upon reasonable request and with approval from the institutional ethics committee.
