# Supplementary material for: Long‐term predictors of seizure outcome after anterior temporal lobectomy in unilateral hippocampal sclerosis: A 281‐patient cohort with mean 10‐year follow‐up
Source: Epileptic Disord. 2025 Nov 24;28(1):84–96. doi: 10.1002/epd2.70139 (PMC12964176; doi:10.1002/epd2.70139)
Supplement: Supplementary file 1 — Appendix S1. [file EPD2-28-84-s001.docx]

**TEST YOURSELF**

**Answers**

1. C

2. C

3. B
